# Supplementary material for: Acceptance and compliance with micronutrient powder (MNP) among children aged 6–23 months in northern Nigeria
Source: PLOS Glob Public Health. 2022 Oct 17;2(10):e0000961. doi: 10.1371/journal.pgph.0000961 (PMC10022258; doi:10.1371/journal.pgph.0000961)
Supplement: S1 Dataset — (ZIP) [file pgph.0000961.s008.zip › S8_Dataset/Direct Observations.zip!/Direct Observations/230605_A_GA_AS_1_DO.pdf]

1.1.1 Instrument F –Direct Observation Form

<<Instrument F Direct Observation Form>>
**PI Name:** PragyaMathema,Stephen Kodish<br>**Study Title:** Formative research to develop an infant and young child feeding (IYCF) intervention with micronutrient powder (MNP) for children aged 6 – 23 months<br>**PI Version/Date:** Version1.0/January 24, 2015

Date: _June 23, 2015_____________Location: _ Ganye 2 community,GANYE, LGA__________

Data Collector: Antiya Sanda____Participant ID: _A house near a forest

Main goals of this direct observation are to answer the following questions:<br><br>1. In this household, what would be the best way(s) to give nutritional supplements to young children? Why?<br><br>2. In this household, describe the food sharing that you observe throughout the meal. Are there any predictable patterns?<br><br>3. In this household, who is favored and how is this favoritism expressed during meal preparation and eating?

(TEAR OFF AT PERFORATED DOTTED LINE BELOW)

PARTICIPANT ID: __

Household Composition Table (Fill out as you do data collection)

INITIALS	SEX (M/F)	AGE (estimated)	Relationship to household	Description
TB	F	29yrs	Mother	A tall slim woman in her late 20's
SM	M	7 months	Baby boy	A small baby boy
TG	F	16 yrs	Cousin	A teenage girl , she's the cousin of the wife
BS	M	32 yrs	Father	A black short man wearing black suit
BY	M	25 yrs	Neighbour	A black young dude wearing a black jacket
TS	F	10 yrs	Neighbour	A tall slim girl from the neighbouring house

PARTICIPANT ID: __________

TIME	ACTOR INITIALS	RECIPIENT INITIALS	WHAT HAPPENED	FOOD	Reactivity
8.00	SM		Sleeping		
8:05	SM		Sleeping		
8:05	SM		Sleeping		
8:10	SM		Sleeping		
8:15	SM		Sleeping		
8:20	SM		Sleeping		
8:25	SM		Sleeping		
8:30	SM		Sleeping		
8:35	SM		Sleeping		
8:40	SM		Wakes from sleep and crying		
08:45	SM		Crying		
08:50	TB	SM	Breastfeeding	Breast milk	
08:55	TB	SM	Breastfeeding	Breast milk	
09:00	TB	SM	Breastfeeding	Breast milk	
09:05	TB	SM	Breastfeeding	Breast milk	

TIME	ACTOR INITIALS	RECIPIENT INITIALS	WHAT HAPPENED	FOOD	Reactivity
09:10	TB	SM	Breastfeeding	Breast milk	
09:15	TB	SM	Breastfeeding	Breast milk	
09:20	TB	SM	Breastfeeding	Breast milk	
09:25	TB	SM	Carry her child on the back		
09:30	TB	SM	Carry her child on the back		
09:35	TB		Brings two flax from outside		
09:40	TB	SM	Still carrying her child on the back		
09:45	TB	SM	Trying to put her child to sleep		
09:55	SM		Still crying		
10:00	SM		Start sleeping		
10:05	TB		Lay her child on the bed		
10:10	TB		Starts sweeping the compound		
10:15	TB		Continues with sweeping		
10:20	TB		Sweeping		
10:25	TB		Starts washing some dirty plates		
10:30	TB		Washing plates		
10:35	TB		Washing plates		
10:40	TB		Start rinsing the plates		
10:45	TB		Rinsing the plates		
10:50	TB		Rinsing the plates		
10:55	TB		Rinsing some big bowls		
11:00	TB		Rinsing some big bowls		
11:05	TB		Start fetching water		
11:10	TB		Fetching water		
11:15	TB		Start sweeping the entire house		
11:20	TB		Continues to sweep the house		
11:25	TB		Start rinsing water pot cover		
11:25	TB		Rinsing water pot cover		
11:30	TB		Brings her child's dirty clothes outside		
11:35	TB		Brings detergent outside		
11:40	TB		Starts washing her child's dirty		

TIME	ACTOR INITIALS	RECIPIENT INITIALS	WHAT HAPPENED	FOOD	Reactivity
			clothes with the detergent		
11:45	TB		Washing her child's clothes		
11:50	TB		Washing her child's clothes		
11:55	TB		Start rinsing the baby's clothes		
12:00	TB		Rinsing the baby's clothes		
12:05	TB		Starts drying the baby's clothes on the dryer		
12:10	TB		Drying her baby's clothes on the dryer		
12:15	TB		Brings her child from the room		
12:20	TB	SM	Start breastfeeding baby	Breast milk	
12:25	TB	SM	Breast feeding baby	Breast milk	
12:30	SM		Starts crying		
12:35	TB	SM	Carry her child on the back		
12:40	BS		Walks in with a can of palm oil	Palm oil	
12:45	BS		Picks a hoe and leaves for the farm, it rained all through the morning so he couldn't leave earlier		
12:46	TB		Brings a hot water flask	Water	
12:47	TB		Brings a bottle of water	Water	
12:48	TB		Brings a cup and spoon		
12:49	TB		Brings her son from the room		
12:50	TB		Opens the hot water in the flask	Water	
12:51	TB		Opens bottle water	Water	
12:52	TB		Pours water into feeding bottle	Water	
12:53	TB		Pours hot water into an empty cup	Water	

TIME	ACTOR INITIALS	RECIPIENT INITIALS	WHAT HAPPENED	FOOD	Reactivity
12:54	TB		Brings a container of cerelac	Cereals	
12:55	TB		Fetches 3 spoons of cerelac into the cup of hot water	Hot water	
12:56	TB		Mixes cerelac with hot water using a spoon	Cereals	
12:57	TB		Uses a spoon to taste the hot water mixed with cerelac	Cereal and water	
12:58	TB		Pours a little cold water on the cerelac	Water and cerelac	
12:59	TB		Mixes it to blend	Water and cereal	
01:00	TB		Allows it to get cold	Water and cereals	
01:01	TB		Waiting for the cerelac	Cereal and water	
01:02	TB		Still mixing the cerelac	Cereal	
01:03	TB		Brings MNP	MNP	
01:04	TB		Trying to unseal the MNP	MNP	
01:05	TB		Bring a scissors		
01:06	TB		Use scissors to cut off the MNP seal	MNP	
01:07	TB		Pours MNP into the cup of cerelac	MNP and Cerelac	
01:08	TB		Mixing the MNP and the cerelac to blend	MNP and cereal	
01:09	TB		Still mixing the MNP	MNP and Cereal	
01:10	TB	SM	Brings a beep and wear it on the neck of the child		
01:11	TB	SM	Start feeding her son with MNP mixed in cerelac but did not have taste		

TIME	ACTOR INITIALS	RECIPIENT INITIALS	WHAT HAPPENED	FOOD	Reactivity
01:13	TB	SM	Continues feeding her child after she has seen that the MNP has blended well	MNP and cereal	
01:14		SM	Opens his mouth and wait for his mother to feed him		
01:15	TB	SM	Feeding her son	MNP and Cereal	
01:16	TB	SM	Feeding her son	MNP and Cereal	
01:17	TB	SM	Feeding her son and singing for him at the same time	MNP and Cereal	
01:18	TB	SM	Feeding her son slowly	MNP and Cereal	
01:19	TB	SM	Feeding her son slowly	MNP and Cereal	
01:20	TB	SM	Feeding her son slowly	MNP and Cereal	
01:21	TB	SM	Feeding her son still using a cup and spoon	MNP and Cereal	
01:22	TB	SM	Gives her child little bottle water to drink	Water	
01:23	TB	SM	Continues feeding her child with MNP mixed cerelac	MNP and Cereal	
01:24	TB	SM	Continues feeding her child	MNP and Cereal	
01:25	TB	SM	Gives her child little bottle water to drink	Water	
01:26	TB	SM	Continues feeding her child	MNP and Cereal	
01:27	TB	SM	Finished all the MNP mixed into cerelac portion	MNP and	

TIME	ACTOR INITIALS	RECIPIENT INITIALS	WHAT HAPPENED	FOOD	Reactivity
				Cereal	
01:28	TB	SM	Cleans the face of her child with a beep		
01:29	TB	SM	Gives him water to drink	Water	
01:30	SM		Start crying		
01:31	TB	SM	Start playing with her child on her lap		
01:32	TB	SM	Carry her child on her back		
01:33	TB		Takes empty cup to rinse		
01:34	TB		Rinsing the cup she fed her child with		
01:35	TB		Gives her child to her cousin		
01:40	TG	SM	Playing with the child in the compound		
01:41	TB		Goes to the kitchen		
01:42	TB		Carry a pot and pours water into it	Water	
01:43	TB		Light the stove		
01:44	TB		Place a pot on the burning stove		
01:45	TB		Cover the pot with water inside	Water	
01:46	TB		Brings an already prepared pot of soup from the second kitchen	spinach	
01:47	TB		Keeps the pot by the side of the stove	Spinach	
01:48	TB		Brings maize flour and a sieve		
01:49	TB		Brings another flat plate		
01:49	TB		Start sieving maize on the flat plate	Maize	
01:49	TB		Continues to sieve maize flour	Maize	
01:49	TB		Fetched some maize flour and pours into a bowl with cold water	Maize and water	
01:49	TB		Start mixing the maize flour with cold water faster to avoid lumps	Maize and water	
01:50	TB		Opens the pot with water on the stove	Water	
01:51	TB		Pour mixed maize flour with cold water into boiling water	Maize and water	
01:52	TB		Removes the stick and keep it aside		
01:53	TB		Cover the pot		
01:54	TB		Bring a food warmer		

TIME	ACTOR INITIALS	RECIPIENT INITIALS	WHAT HAPPENED	FOOD	Reactivity
01:56	TB		Opens the pot on the fire		
01:57	TB		Pours maize flour and begin to mix faster	maize	
01:58	TB		Mixing the maize flour into tuwo	maize	
01:59	TB		Removes the stick and covers the pot		
02:00	TB		Brings a serving spoon		
02:01	TB		Opens the pot on the fire		
02:02	TB		Start serving tuwo into the food warmer	Maize	
02:03	TB		Continue to serve the tuwo	Maize	
02:04	TB		Serves all the tuwo into the food warmer	Maize	
02:05	TB		Carry pot and pour water into it		
02:06	TB		Reduce the flame of the stove		
02:07	TB		Puts the pot of soup on the stove	Spinach	
02:08	TB		Opens the pot of soup	Spinach	
02:09	TB		Stirs the soup	Spinach	
02:10	TB		Use a spoon to taste the soup	Spinach	
02:11	TB		Drop the spoon and cover the pot		
02:12	TB		Standing and waiting for the soup to steam	Spinach	
02:13	TB		Bring down the pot of soup from the stove	Spinach	
02:14	TB		Sprinkles little water to quench the stove		
02:15	TB		Carry the food warmer to a corner	Maize	
02:20	TQ,SM		Playing together in the compound		
02:25	TB		Start cleaning an umbrella		
02:30	TB		Cleaning an umbrella using a rag		
02:35	TB		Bring dirty plate from the kitchen		
02:40	TB		Bring cups and jugs to the compound		
02:45	TB		Gathered the dirty plates, cups and jugs in a big bowl		
02:50	TB		Pour water on the dirty plates, cups and jugs		
02:55	TB		Bring out liquid detergent		
03:00	TB		Starts washing the cups, plates and the jugs		
03:05	TB		Continue washing		

TIME	ACTOR INITIALS	RECIPIENT INITIALS	WHAT HAPPENED	FOOD	Reactivity
3:10	TB		Start rinsing the plates and jugs		
03:15	TB		Continues to rinse		
03:20	TB		Start washing all pots		
03:25	TB		Washing all pots		
03:30	TB		Continues to wash the back of the pot using sand and detergent		
03:35	TB		Start rinsing all the pots		
03:40	TB		Takes the dirty plates water outside to pour away		
03:45	TB		Returns with the empty big bowl		
03:50	TB		Sweeping the spot where she washed the plates and pots		
03:55	TB		Starts fetching water in a bucket		
04:00	TB		Goes to the bathroom with a bucket of water		
04:05	TB		Playing alone in the compound while sitting on a mat		
04:10	TB		Comes out of the bathroom		
04:15			Enters a room		
04:20			Comes out of the room well dressed		
04:25	TB	SM	Carry her child on her lap		
04:30	TB	SM	Start breastfeeding	Breast milk	
04:35	TB	SM	Breastfeeding her child	Breast milk	
04:40	TB	SM	Breastfeeding her child	Breast milk	
04:45	TB	SM	Breastfeeding her child	Breast milk	
04:50	TB	SM	Breastfeeding her child	Breast milk	
04:55	TB	SM	Breastfeeding her child	Breast milk	
05:00	TB	SM	Breastfeeding her child	Breast milk	
05:05	TB	SM	Breastfeeding her child	Breast milk	
05:10	TB	SM	Breastfeeding her child	Breast	

TIME	ACTOR INITIALS	RECIPIENT INITIALS	WHAT HAPPENED	FOOD	Reactivity
				milk	
05:15	TB	SM	Breastfeeding her child	Breast milk	
05:20	TB	SM	Breastfeeding her child	Breast milk	
05:25	TB	SM	Breastfeeding her child	Breast milk	
05:30	TB	SM	Carry her child to the room		
05:35	SM		Sleeping		
05:40	SM		Sleeping		
05:45	SM		Sleeping		
05:50	SM		Sleeping		
05:55	TB		Sit on a chair in the compound		
06:00	TB		Sitting and combing her hair		

PARTICIPANT ID: __

Primary Questions (Complete after you complete data collection):

From what you have seen observing THIS HOUSEHOLD, please answer the following questions in detail:

Question	Write your detailed response here
1. In this household, what would be the best way(s) to give nutritional supplements such as an MNP to young children to ensure compliance?<br><br>Why?	The best way to give MNP to the young child should be in the afternoon after the child wakes from his usual nap.<br><br>Because this young child just got introduced to complimentary feeding and he is used to eating semi-solid food only in the afternoon after his nap.
2. In this household, who is favored during meal preparation or feeding?<br><br>How is this favoritism expressed?	Nobody is favoured during meal preparation because there are no children in this household and after the meal is prepared all the food is served in the food warmer for just the husband or wife to fetch when they are hungry.

3. How could we promote the MNP supplement for this family that would attract their attention?<br><br>Who would they listen to?	MNP can be promoted to this family through the mother since she stays all day with the child unlike the father who goes to the farm and stays all day in the farm.<br><br>The young child would listen to the mother more because she understands the child better than anyone else and she is always around the child to play with him and also spend some quality time in his upbringing.
4. In this household, what would be the best ways to promote the MNP but limit how much it would be shared?<br><br>Why?	The best way would be to introduce the MNP during his complimentary feeding which is usually in the afternoon. The tendency that the MNP could be shared is very limited because the young child in this household is the only child with no siblings.<br><br>The mother is well organized woman and there are no older children or children from the neighbourhood that can gain access to where the MNP is stored.
5. What other issues and concerns did you see with this family that are important for us to consider with regard to introduction of an MNP?<br><br>Why?	There are no issues or concerns with the regard to the introduction of MNP in this household.<br><br>This household has enough food, the mother maintains a good hygiene. The young child feeding pattern is okay because he eats all the semisolid food mixed with MNP without rejection. There are no issues.
6. Based on your experience observing this child today, do you believe reactivity negatively impacted your ability to see the food-related behaviors of the child?<br><br>Please explain your answer in detail.	Reactivity did not negatively impact my ability to see any food related behavior.<br>There was no such thing as a reactive behavior in this house.
